# Structure Water-Solubility Relationship in *α*-Helix-Rich Films Cast From Aqueous and 1,1,1,3,3,3-Hexafluoro-2-Propanol Solutions of *S. c. ricini* Silk Fibroin

**DOI:** 10.3390/molecules24213945

**Published:** 2019-10-31

**Authors:** Kelvin O. Moseti, Taiyo Yoshioka, Tsunenori Kameda, Yasumoto Nakazawa

**Affiliations:** 1 Department of Biotechnology and Life Science, Graduate School of Engineering, Tokyo University of Agriculture and Technology, 2-24-16 Naka-cho, Koganei, Tokyo 184-8588, Japan; kelvinmoseti@gmail.com; 2 Silk Materials Research Unit, Institute of Agrobiological Sciences, National Agriculture and Food Research Organization, 1-2 Owashi, Tsukuba, Ibaraki 305-8634, Japan; yoshiokat@affrc.go.jp; 3 National Sericulture Research Centre, Industrial Crops Research Institute, Kenya Agricultural and Livestock Research Organization, P.O. Box 7816-01000 Thika, Kenya

**Keywords:** wild silkworm silk, *α*-helix-rich, ordered aggregated *α*-helix structure, water-resistance, biomaterials

## Abstract

Silk fibroin (SF) produced by the domesticated wild silkworm, *Samia cynthia ricini* (*S. c. ricini*) is attracting increasing interest owing to its unique mechanical properties, biocompatibility, and abundance in nature. However, its utilization is limited, largely due to lack of appropriate processing strategies. Various strategies have been assessed to regenerate cocoon SF, as well as the use of aqueous liquid fibroin (LF_aq_) prepared by dissolution of silk dope obtained from the silk glands of mature silkworm larvae in water. However, films cast from these fibroin solutions in water or organic solvents are often water-soluble and require post-treatment to render them water-stable. Here, we present a strategy for fabrication of water-stable films from *S. c. ricini* silk gland fibroin (SGF) without post-treatment. Aqueous ethanol induced gelation of fibroin in the posterior silk glands (PSG), enabling its separation from the rest of the silk gland. When dissolved in 1,1,1,3,3,3-hexafluoro-2-propanol (HFIP), the SGF-gel gave a solution from which a transparent, flexible, and water-insoluble film (SGF_HFIP_) was cast. Detailed structural characterization of the SGF_HFIP_ as-cast film was carried out and compared to a conventional, water-soluble film cast from LF_aq_. FTIR and ^13^C solid-state NMR analyses revealed both cast films to be *α*-helix-rich. However, gelation of SGF induced by the 40%-EtOH-treatment resulted in an imperfect *β*-sheet structure. As a result, the SGF-gel was soluble in HFIP, but some *β*-sheet structural memory remains, and the SGF_HFIP_ as-cast film obtained has some *β*-sheet content which renders it water-resistant. These results reveal a structure water-solubility relationship in *S. c. ricini* SF films that may offer useful insights towards tunable fabrication of novel biomaterials. A plausible model of the mechanism that leads to the difference in water resistance of the two kinds of *α*-helix-rich films is proposed.

## 1. Introduction

Fiber spun by the domesticated wild silkworm, *Samia cynthia ricini* (*S. c. ricini*), is essentially different from that spun by the mulberry silkworm, *Bombyx mori* (*B. mori*). While the primary structure of *S. c. ricini* silk fibroin (SF) contains repeat poly-L-alanine (PA) sequences [(Ala)_12–13_] [1] that predominantly form a water-soluble *α*-helix structure [2], that of *B. mori* contains a (GAGAGS)_n_ repeat motif [3] that forms the metastable silk I structure [4]. During natural spinning by mature *S. c. ricini* larvae, *α*-helix to *β*-sheet transition occurs and the native silk produced is water-resistant [2,5,6]. Materials (in various morphologies) such as films, gels, nanoparticles, and sponges can be fabricated from *S. c. ricini* SF solutions in water or organic solvents such as 1,1,1,3,3,3-hexafluoro-2-propanol (HFIP) and trifluoroacetic acid (TFA). Due to the difference in primary structure, such biomaterials may exhibit different material and biomedical properties that may be handy in the design and development of novel biomaterials. Therefore, regeneration of *S. c. ricini* cocoon SF is required to obtain solutions for fabrication. However, owing to the unique structure of *S. c. ricini* SF, attempts to use the traditional regeneration protocols for *B. mori* have been unsuccessful. For instance, SF powder [7] and fiber [8] regenerated from *S. c. ricini* cocoons had lower molecular weight (M_W_) and diminished fiber tenacity, respectively, due to hydrolysis resulting from relatively harsh preparative conditions such as degumming temperature. This has limited effective utilization of this unique bioresource [9].

**Scheme 1:**
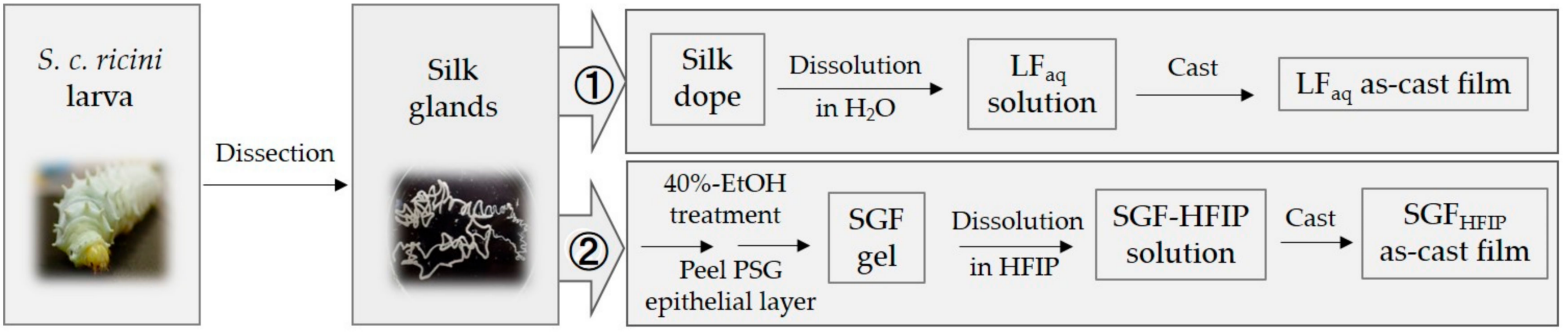
Fabrication of cast films from *Samia cynthia ricini* (*S. c. ricini*) aqueous liquid fibroin (LF_aq_) (route ①) and 1,1,1,3,3,3-hexafluoro-2-propanol (HFIP) silk gland fibroin (SGF_HFIP_) (route ②) solutions.

To overcome this shortcoming, fabrication of biomaterials using liquid fibroin (LF) obtained directly from the posterior silk gland (PSG) of mature *S. c. ricini* larvae has been explored [5,10,11]. As earlier reported, films cast from *S. c. ricini* LF at room temperature (~23 °C), as shown in route ① in Scheme 1 were *α*-helix-rich [12]. In addition, it takes several minutes to collect workable amounts of LF from the PSG of *S. c. ricini*, that is, this fabrication approach is a time-consuming downside, if large-scale applications are desirable. Moreover, the LF-based films are water-soluble [5,10], and require post-treatment to induce water resistance, a desirable property for wet-state applications. *β*-sheet formation, often induced by treatment with aqueous alcohol solutions, and thermal- and water-vapor annealing (refer to Yoshioka et al. [13] and references therein), is the most widely used strategy to render the biomaterials water-resistant. However, such post-treatments often modify inherent desirable properties of the LF-based materials, limiting their applicability [9]. Thus, the need for an alternative fabrication strategy for *S. c. ricini* silk, and indeed other wild silks, is imperative, owing to their attractive mechanical properties, biocompatibility, biodegradation, and abundant yields in nature [9,14].

Recently, we developed a mild approach to fabricate high-molecular-weight, water-resistant cast films directly, that is, without the need of the time-consuming steps as well as post-product treatment, from *B. mori* silk gland fibroin (SGF) [13]. However, the cast film obtained was *β*-sheet-rich and very long dissolution durations (>60 days) were required to afford *α*-helix-rich as-cast films. If this fabrication approach is successfully applied to *S. c. ricini* SGF, one can expect to obtain *α*-helix-rich as-cast films because of the fibroin proteins’ characteristic PA repeat sequences.

In the current study, we successfully applied a similar strategy in the fabrication of *S. c. ricini* SGF cast films as summarized in route ② in Scheme 1. As earlier reported for *B. mori* SF [13], preliminary studies and the current study also revealed 40% ethanol/water solution (v/v; 40%-EtOH) to be the optimum concentration that gave a gel of *S. c. ricini* SGF that was easy to separate from the rest of the silk gland, and soluble in HFIP. The solution of the gel (herein, SGF-gel) in HFIP was transparent. Films were cast from this solution at room temperature and compared to the conventional ones cast from an aqueous solution liquid fibroin (LF_aq_) prepared as earlier described [12].

The structure and properties of the cast films were systematically investigated in detail by thermal analyses, ^13^C solid-state NMR spectroscopy, Fourier transform infrared spectroscopy (FTIR), wide-angle X-ray diffraction (WAXD), high performance liquid chromatography (HPLC), and gel permeation chromatography (GPC). Our results revealed a structure water-solubility relationship in the LF_aq_ and SGF_HFIP_ *S. c. ricini* as-cast films. On the basis of these experimental results, a plausible model of the mechanism leading to water resistance of the SGF_HFIP_ as-cast film is proposed.

## 2. Results and Discussion

### 2.1. Structure and Properties of the LF_aq_ and SGF_HFIP_ As-Cast Films

**Figure 1:**
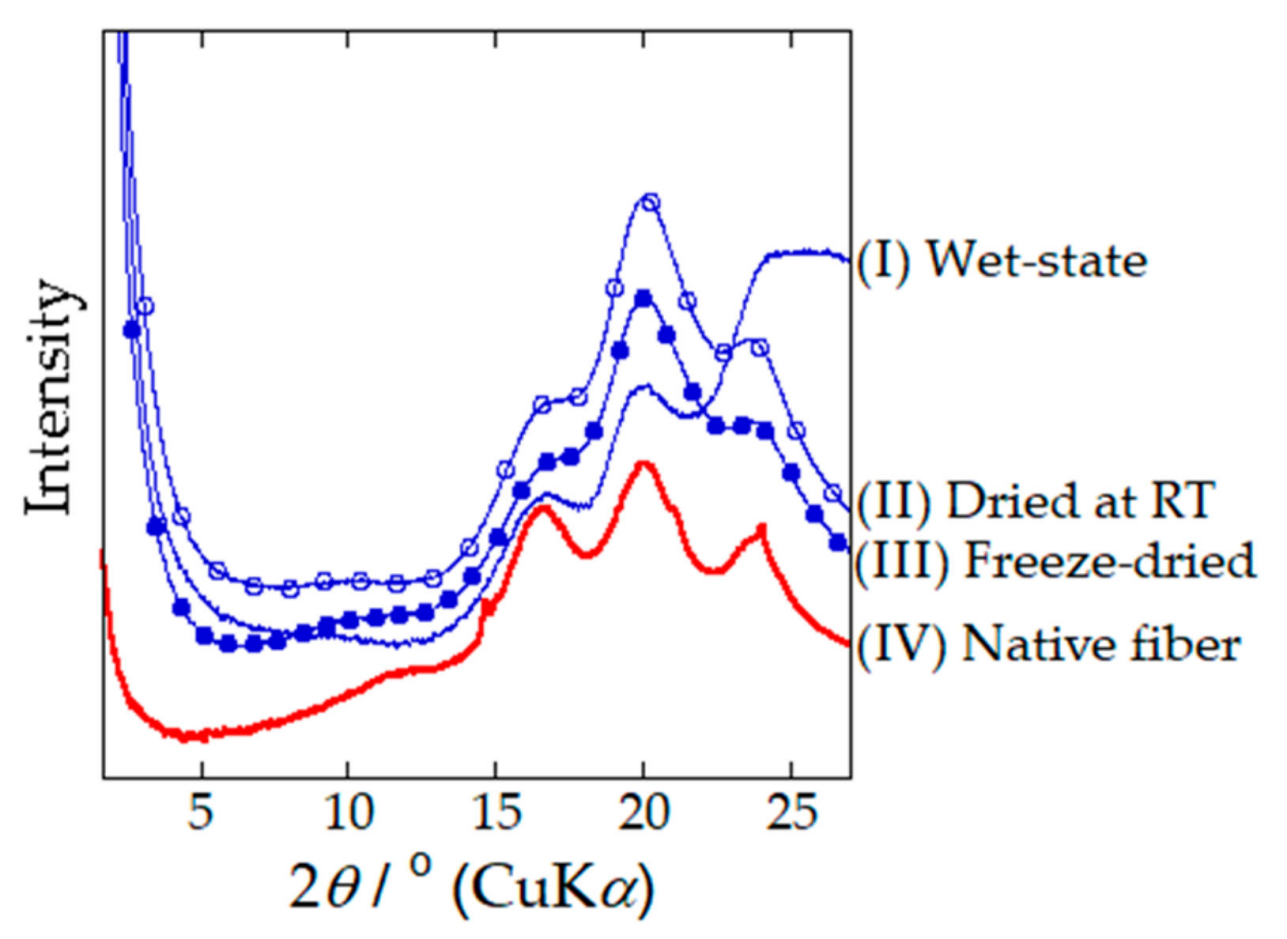
Wide-angle X-ray diffraction (WAXD) 2*θ*-profiles of *S. c. ricini* silk gland fibroin gel (SGF-gel) in the wet- and dry-states. Profile I is for the wet-state gel (in 40%-EtOH), II is for the gel dried at room temperature, III is for the freeze-dried gel, and IV is for native fiber.

Immersion of freshly extracted *S. c. ricini* silk glands in 40%-EtOH induced *β*-sheet formation of SGF in the lumen of the PSG within a few hours (~3 h; visual observation) and peeling the gland epithelium gave a transparent SGF-gel. WAXD 2*θ*-profiles of the SGF-gel in the wet- (40%-EtOH) and dry-states were as shown in Figure 1. Compared to the *β*-sheet-rich profile of *S. c. ricini* native fiber (see profile IV), the SGF-gel already exhibited a *β*-sheet profile in the wet-state (profile I). However, this profile was somehow distorted, probably due to a high amount of adsorbed EtOH and bulk water in the SGF-gel. By drying the SGF-gel at room temperature or freeze-drying, the EtOH and bulk water were removed or substantially reduced and the profiles of these two dry-states (see profiles II and III) were evidently *β*-sheet-rich.

To evaluate the purity of the SGF-gel, its Mw distribution was evaluated by gel permeation chromatography (GPC) and compared to that of a conventional film cast from LF (LF_aq_ as-cast film) prepared by dissolution of silk dope obtained directly from the silk glands of mature *S. c. ricini* larvae in water. The differential Mw distribution of the SGF-gel was essentially similar to that of the LF_aq_ as-cast film as shown in Figure S1. This demonstrates good separation of the SGF-gel from the rest of the silk gland (epithelium and sericin layers). 

HFIP is known to be an excellent solvent for *B. mori* SF in an amorphous state [15]. It causes negligible molecular weight damage [15,16], and induces and stabilizes the *α*-helical conformation [17,18]. On the other hand, once SF forms the *β*-sheet crystal, it hardly dissolves, even in HFIP [19]. However, the SGF-gel which forms an imperfect *β*-sheet structure when treated with 40%-EtOH [13], dissolved in HFIP within 2 h at 23 ± 2 °C under mild agitation to give a transparent solution. FTIR results of films cast from solutions prepared by dissolution of the SGF-gel in HFIP (SGF_HFIP_ as-cast films) at 23 ± 2 °C for different durations (between 1.25 and 24 h) were as shown in Figure S2. These results suggest that the dissolution of the SGF-gel in HFIP does not depend on the dissolution duration. Further, the amino acid composition analyses of the film cast from a solution of the SGF-gel in HFIP for 2 h, SGF_HFIP_ (a), and the LF_aq_ as-cast film were as shown in Table 1. The molar ratios of Ser residues in the SGF_HFIP_ (a) and LF_aq_ as-cast films were comparable. As allredy mentioned in the Mw distribution, these results further demonstrate the successful separation of *S. c. ricini* SGF from the silk glands in a form that is easy to dissolve in HFIP.

**Figure 2:**
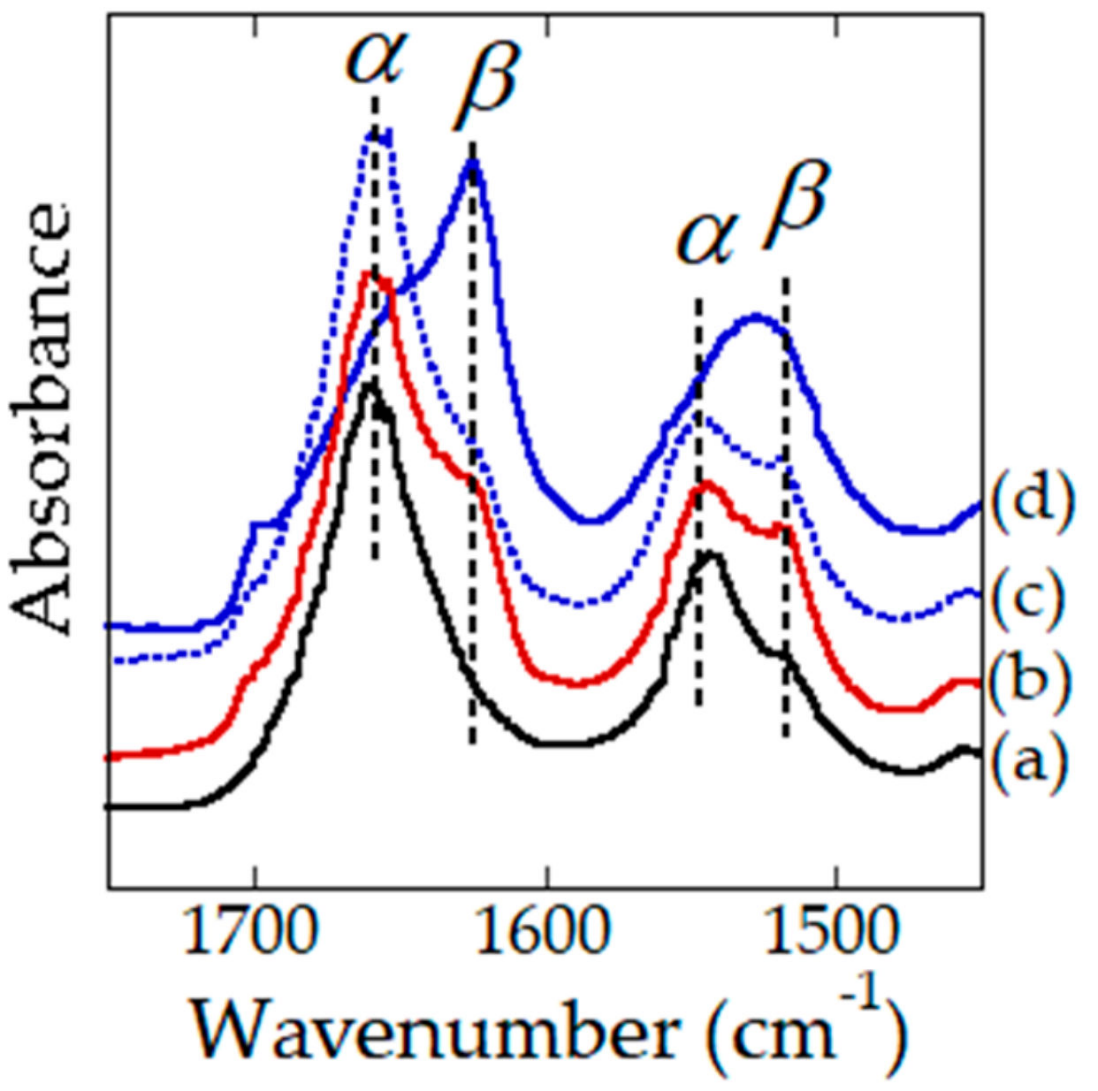
FTIR spectra of films cast from silk fibroin (SF) solutions obtained by dissolution of 40%-EtOH-solidified *Bombyx mori* (*B. mori*) SGF for 10 days (spectrum **d**) and 90 days (spectrum **c**), and *S. c. ricini* SGF for 12 h in HFIP (spectrum **b**) at 23 ± 2 °C. Spectrum (**a**) is that of the film cast from an aqueous solution of *S. c. ricini* LF.

**Figure 3:**
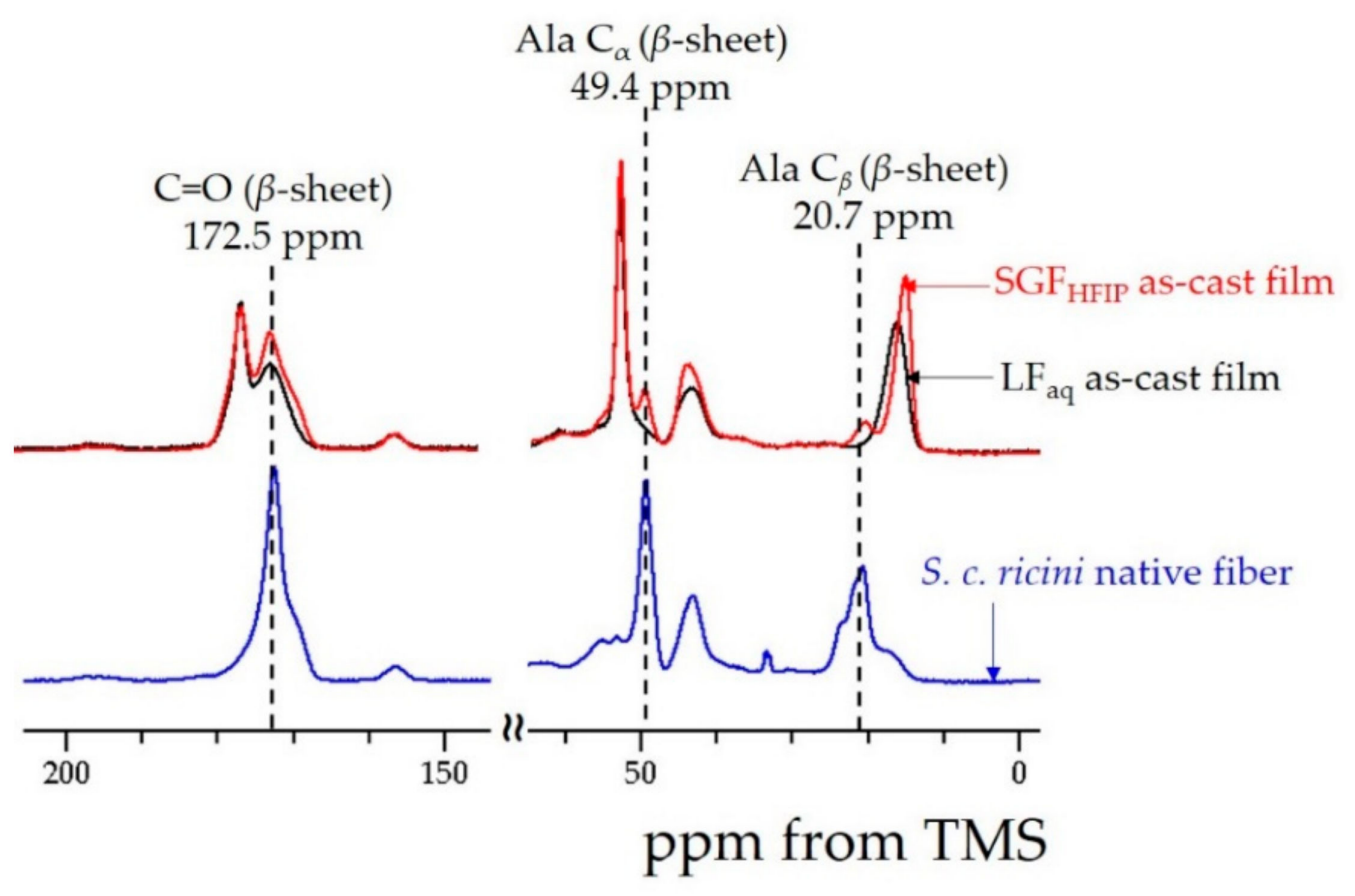
^13^C solid-state NMR spectra in the ranges 0 to 55 and 150 to 200 ppm of *S. c. ricini* native fiber (spectrum in blue), and LF_aq_ (spectrum in black) and SGF_HFIP_ (spectrum in red) as-cast films.

Similar to the *S. c. ricini* LF_aq_ as-cast film reported in our earlier study [12], the SGF_HFIP_ as-cast film was transparent and flexible as shown in Figure S3. FTIR results of the LF_aq_ and SGF_HFIP_ as-cast films were as shown in in Figure 2a,b. Both films were *α*-helix-rich as seen in the amide I and II bands of their spectra. These results were well supported by ^13^C solid-state NMR results shown in Figure 3. Unlike the case of *B. mori* SGF-gel in our recent report in which long dissolution durations (>60d) were necessary to obtain *α*-helix-rich as-cast films (Figure 2c,d) [13], a short-dissolution duration was sufficient for *S. c. ricini* SGF-gel. This was speculated to result from the rich content of Ala residues (~45 mol%) in the SF [1], an intrinsically *α*-helix-stabilizing amino acid [20]. Moreover, the stability of the *α*-helical conformation has also been ascribed to a winding phenomenon typical to the N- and C-termini of the PA sequences of *S. c. ricini* SF [21].

The LF_aq_ as-cast film dissolved almost immediately when placed on a water surface, whereas the SGF_HFIP_ as-cast one remained afloat for more than two weeks (see Figure S4). The Mw of SF has been reported to influence the solubility of SF in water [22]. However, the differential Mw distributions of the SGF-gel and LF_aq_ as-cast film were essentially similar as already mentioned (Figure S1). This implies that the Mw did not possibly contribute to the difference observed in water resistance. In our earlier study, the amide I region of the FTIR spectrum of the LF_aq_ as-cast film was assigned to the *α*-helical conformation, whereas the amide II region was speculated to contain some amount of *β*-sheet or other components [12]. In this study, detailed comparative examination of the FTIR spectra of the LF_aq_ and SGF_HFIP_ as-cast films confirmed a small but highly reproducible: The SGF_HFIP_ as-cast film clearly had some *β*-sheet content in the amide I and II regions. This difference was also discernible in the NMR results as shown in the chemical shifts highlighted in Figure 3.

### 2.2. Ordered α-Helix Structure in the SGF_HFIP_ As-Cast Film

**Figure 4:**
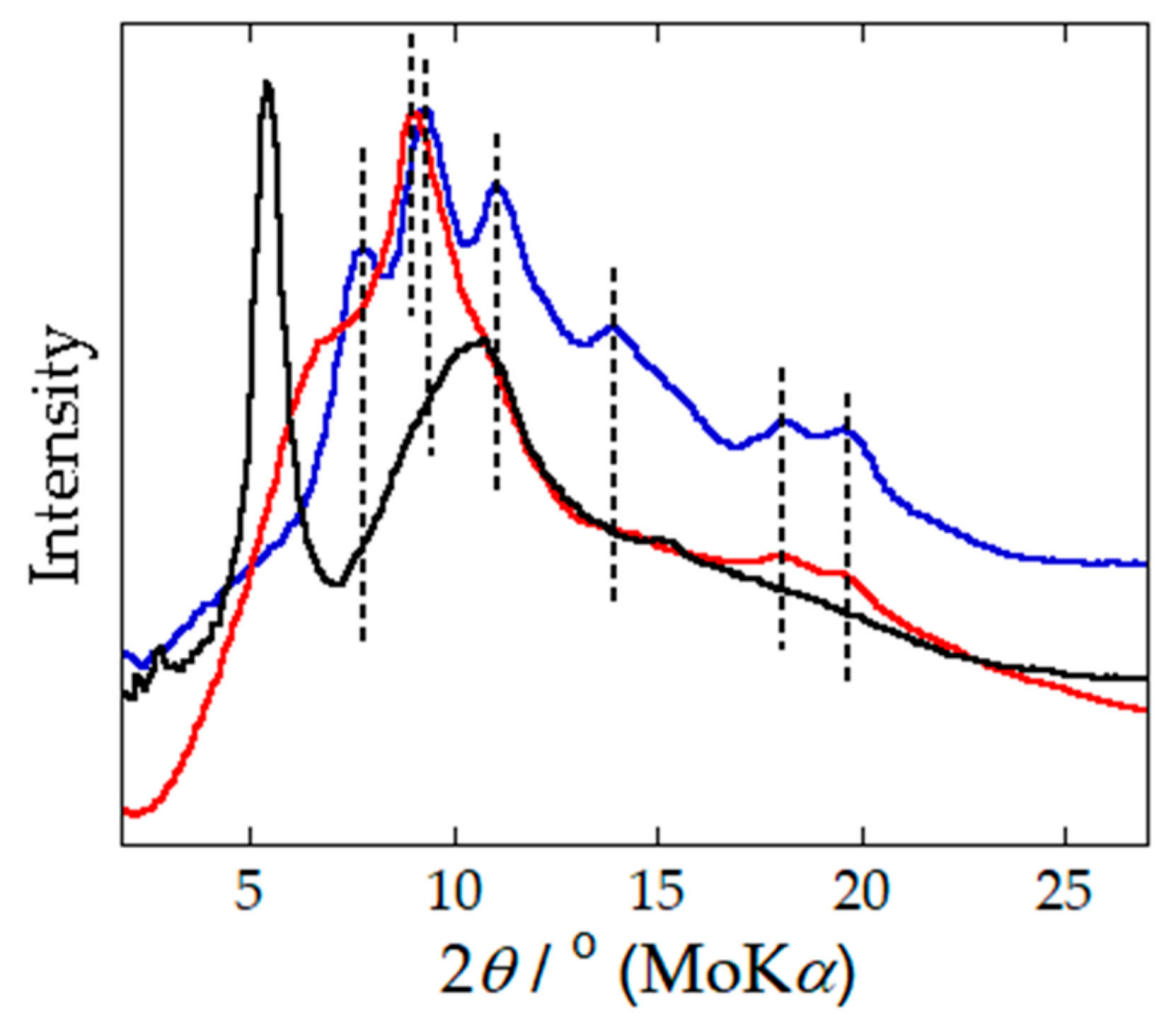
WAXD 2*θ*-profiles of the dry SGF-gel (blue), and LF_aq_ (black) and SGF_HFIP_ (red) as-cast films. The black, dotted lines indicate peaks assigned to the *β*-sheet structure.

WAXD 2*θ*-profiles of the LF_aq_ and SGF_HFIP_ as-cast films compared to that of a *β*-sheet-rich dry SGF-gel are shown in Figure 4. Weak, but distinct and reproducible wide-angle scattering peaks (obtained by Mo-K*α* 0.7083 Å radiation) assigned to the *β*-sheet conformation were observed at 2*θ* = 18.2° (2.3 Å: corresponding to the *β*_210_ reflection) and 2*θ* = 19.7° (2.1 Å: corresponding to the *β*_103_ reflection) in the profile for the SGF_HFIP_ as-cast film, but absent in that of the LF_aq_ as-cast film. This confirmed the FTIR and NMR results regarding the presence of some *β*-sheet structure in the SGF_HFIP_ as-cast film. On the other hand, a sharp and intense peak at 2*θ* = 5.5° (corresponding to 7.4 Å) was observed for the LF_aq_ as-cast film but absent in the dry SGF-gel. This peak implied the existence of long-range order, and was clarified to correspond to ordered aggregation of the *α*-helix structure [12].

**Figure 5:**
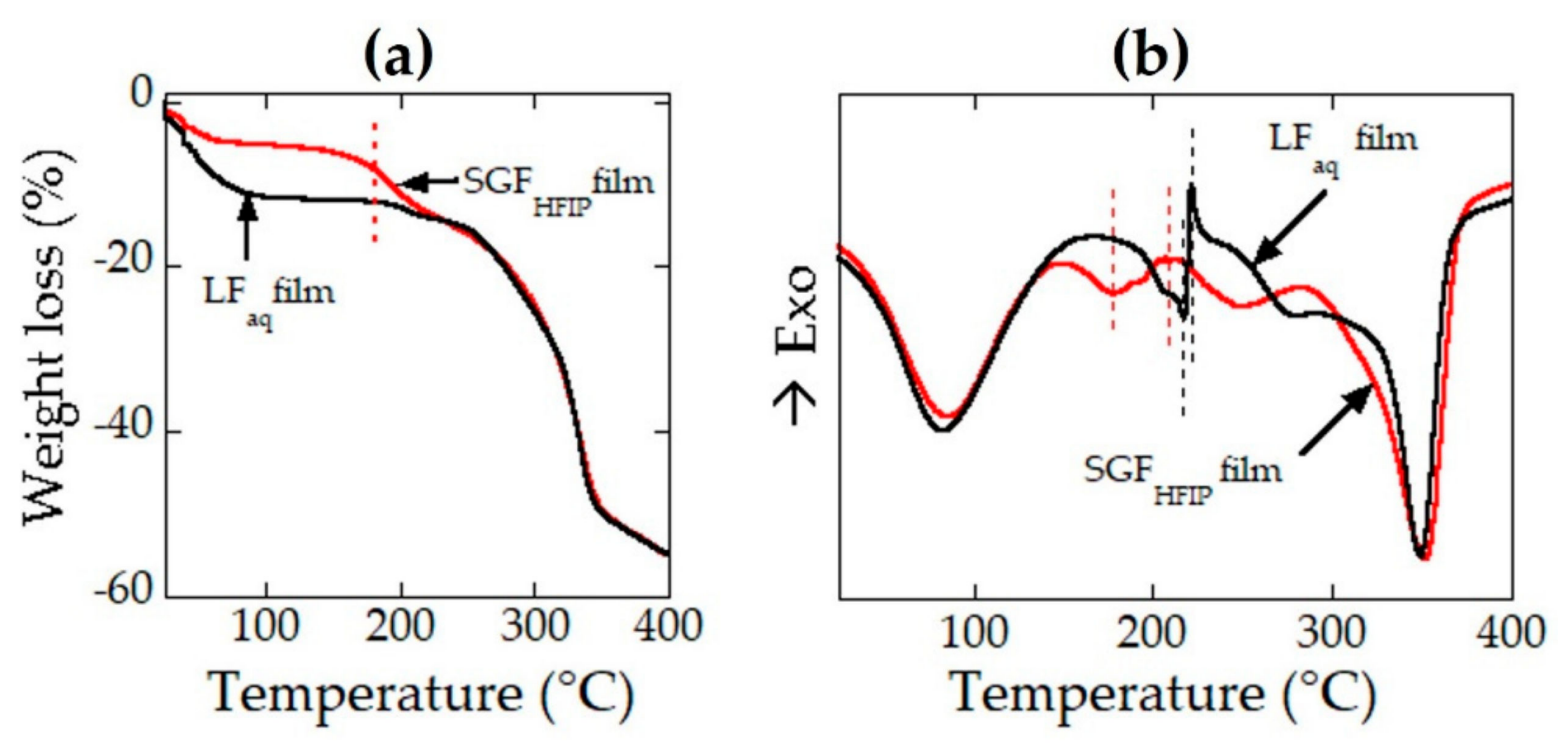
Thermal gravimetry (TG) (**a**) and differential scanning calorimetry (DSC) (**b**) profiles of *S. c.* *ricini* LF_aq_ (profile in black) and SGF_HFIP_ (profile in red) as-cast films.

Thermal gravimetry (TG) and differential scanning calorimetry (DSC) profiles of the LF_aq_ and SGF_HFIP_ as-cast films were as shown in Figure 5. In the TG profiles, a significant loss in weight was detected at ~180 °C in the profile of the SGF_HFIP_ as-cast film (indicated by a red dotted line in Figure 5a), but absent in that of the LF_aq_ as-cast film. In an earlier study on *B. mori* SGF, residual HFIP molecules were confirmed to form an *α*-helix-HFIP complex [17,18] that was stable up to ~140 °C, which is higher than the boiling point of HFIP (~58.2 °C) [17]. This weight loss was considered to be due to the loss of residual HFIP, which was detected by FTIR (see Figure S5) in *S. c. ricini* SGF_HFIP_ as-cast film, due to a similar phenomenon.

**Figure 6:**
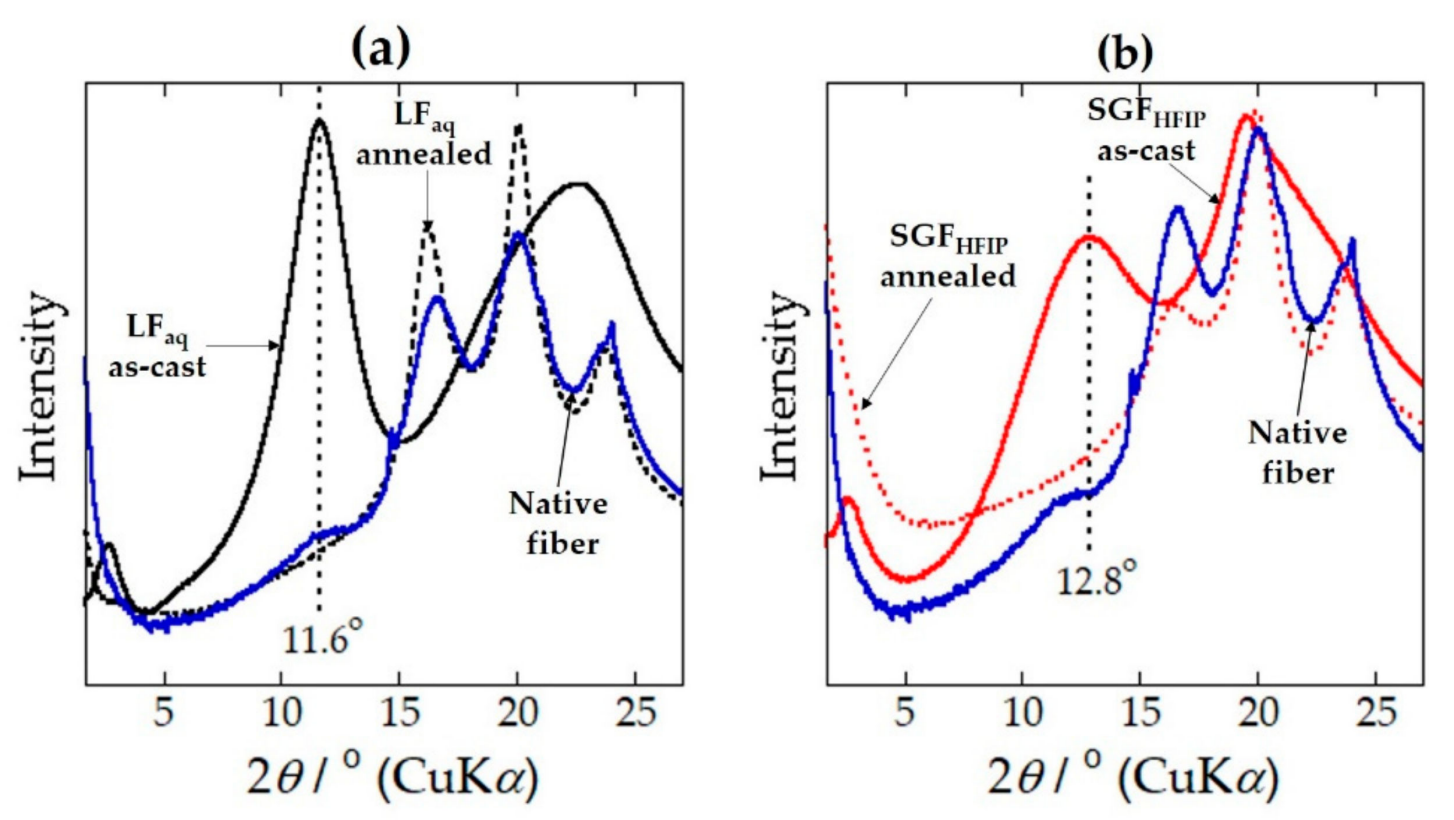
WAXD 2*θ*-profiles of *S. c. ricini* LF_aq_ (**a**, black) and SGF_HFIP_ (**b**, red) films, and native fiber (blue). The solid- and dotted-line profiles correspond to the as-cast and heat-treated (220 °C, 1 min) films, respectively.

In the DSC profiles, the exothermic peak of the SGF_HFIP_ as-cast film was broader and was detected at a slightly lower peak temperature (~200 °C) compared to the LF_aq_ as-cast film (Figure 5b). WAXD analyses shown in Figure 6 (and discussed thereafter) suggested the shift and broadening of this peak to be due to closer packing and wider distribution, respectively, of the hexagonal packing of the *α*-helical domains.

WAXD 2*θ*-profiles of native fiber (same profile shown in Figure 1), as-cast and heat-treated LF_aq_ and SGF_HFIP_ films are shown in Figure 6. As earlier reported, *α*-helices in the LF_aq_ as-cast film were confirmed to self-assemble into an ordered aggregated *α*-helix structure [12]. This is well consistent with an earlier study by atomic force microscopy (AFM) in which *S. c. ricini* fibroin molecules were shown to self-assemble via electrostatic interactions to form highly ordered structures stabilized by intramolecular hydrogen bonds [23]. The profile of the SGF_HFIP_ as-cast film (Figure 6b) had features essentially similar to those of the as-cast LF_aq_ film (Figure 6a). However, the position of the (10-10) peak in the profile of the LF_aq_ as-cast film (detected at 2*θ* = 11.6°; corresponding to *d* = 7.6 Å) (Figure 6a) became broad and shifted to a slightly wide-angle (2*θ* = 12.8°; corresponding to *d* = 7.0 Å) in the profile of the SGF_HFIP_ as-cast film (Figure 6b). The shift in peak position and peak broadening are considered to be due to the closer packing but wider distribution of the ordered aggregation state of the *α*-helices, as already mentioned in the DSC results. Both profiles showed a clear structural transition to the *β*-sheet structure due to heat treatment at 220 °C for 1 min (Figure 6a,b).

### 2.3. Structural Origin of the Water-Resistance Property of the SGF_HFIP_ As-Cast Film

To clarify the structural origin of the water resistance property of the SGF_HFIP_ as-cast film, rectangular portions of the water-soluble LF_aq_ as-cast film were placed in a container saturated with HFIP gas at 23 ± 2 °C for 20 min. The shape of the HFIP-gas-treated film was not distorted, but the peaks assigned to HFIP were detected in the FTIR spectrum of the film. This indicated possible interaction between HFIP and the *α*-helices in the LF_aq_ as-cast film. However, the film remained water-soluble. This observation further suggested that the aggregated state of the *α*-helices in the film treated with HFIP gas was not affected. Hence, immediately the film was immersed in water, HFIP dissolved, its structure became similar to that of the LF_aq_ as-cast film, and it dissolved. 

**Figure 7:**
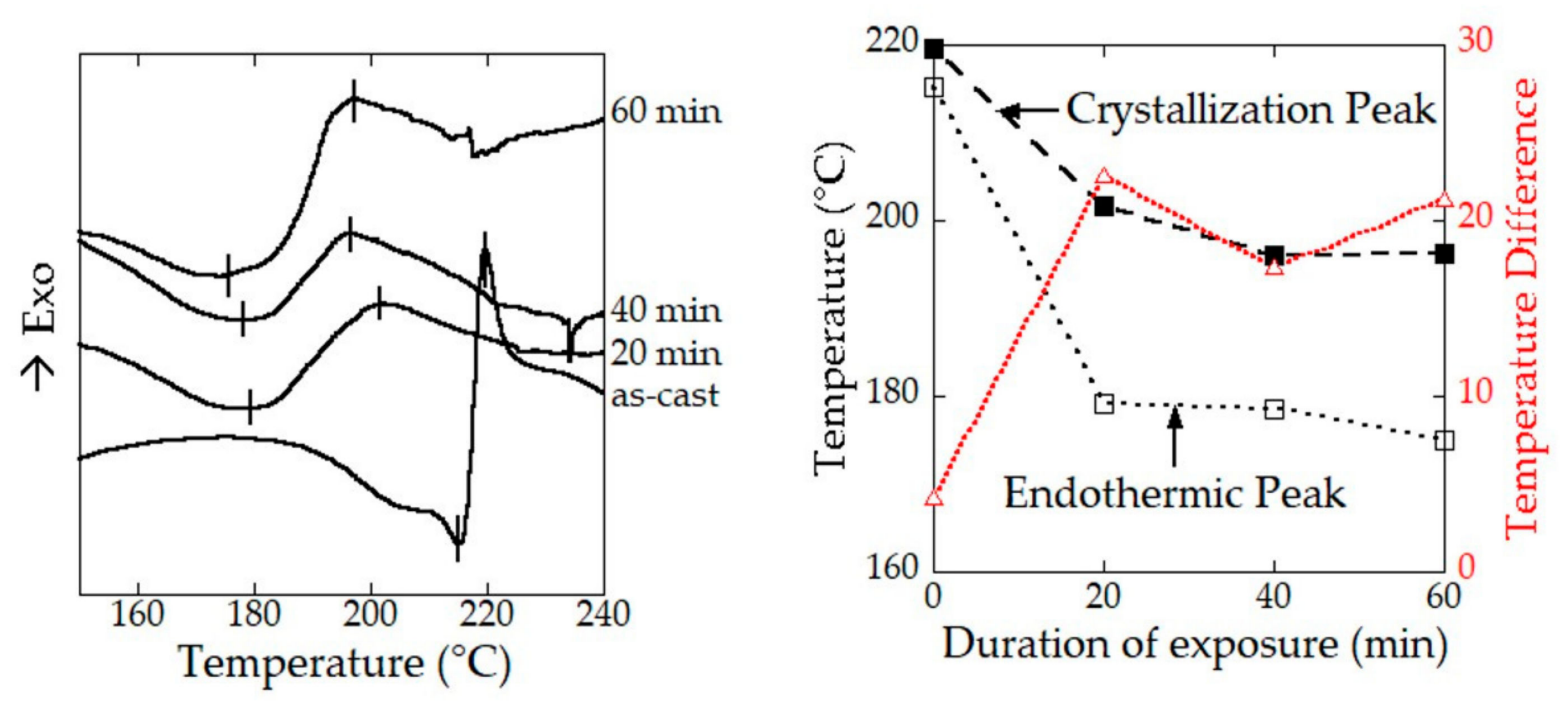
DSC *β*-sheet crystallization peaks of the *S. c. ricini* LF_aq_ film treated with HFIP gas at 23 °C for 20, 40, and 60 min. The panel on the right is a plot of the *β-*sheet crystallization peak temperatures against the duration of exposure to HFIP gas.

The sharp *β*-sheet crystallization peak detected in the LF_aq_ as-cast film broadened and was detected at a lower temperature in the DSC profile of the HFIP-gas-treated film as shown in Figure 7. Since surface adsorbed HFIP would easily evaporate during the DSC scan, this result confirms the interaction between HFIP and the ordered aggregated *α*-helices. To account for the peak shift and broadening detected in the profile of the film treated with HFIP gas, the gradual increase in temperature during the DSC scan was considered to increase the interaction between fibroin and HFIP molecules, eventually resulting in a structure similar to that of the SGF_HFIP_ as-cast film. This implies that HFIP complexed with the *α*-helices, but the molecular movements in the solid-state are relatively low, hence the overall aggregated state is retained. This reasonably accounts for the broadening and shift of the endothermic peak even when the LF_aq_ as-cast film was treated with HFIP gas under mild conditions. Further exposure of the LF_aq_ as-cast film to HFIP gas for longer durations and at higher temperatures gave similar results (see Figure 7, Figure S6). However, higher temperatures (>50 °C) and longer exposure durations resulted in deformation and eventual dissolution of the film.

**Figure 8:**
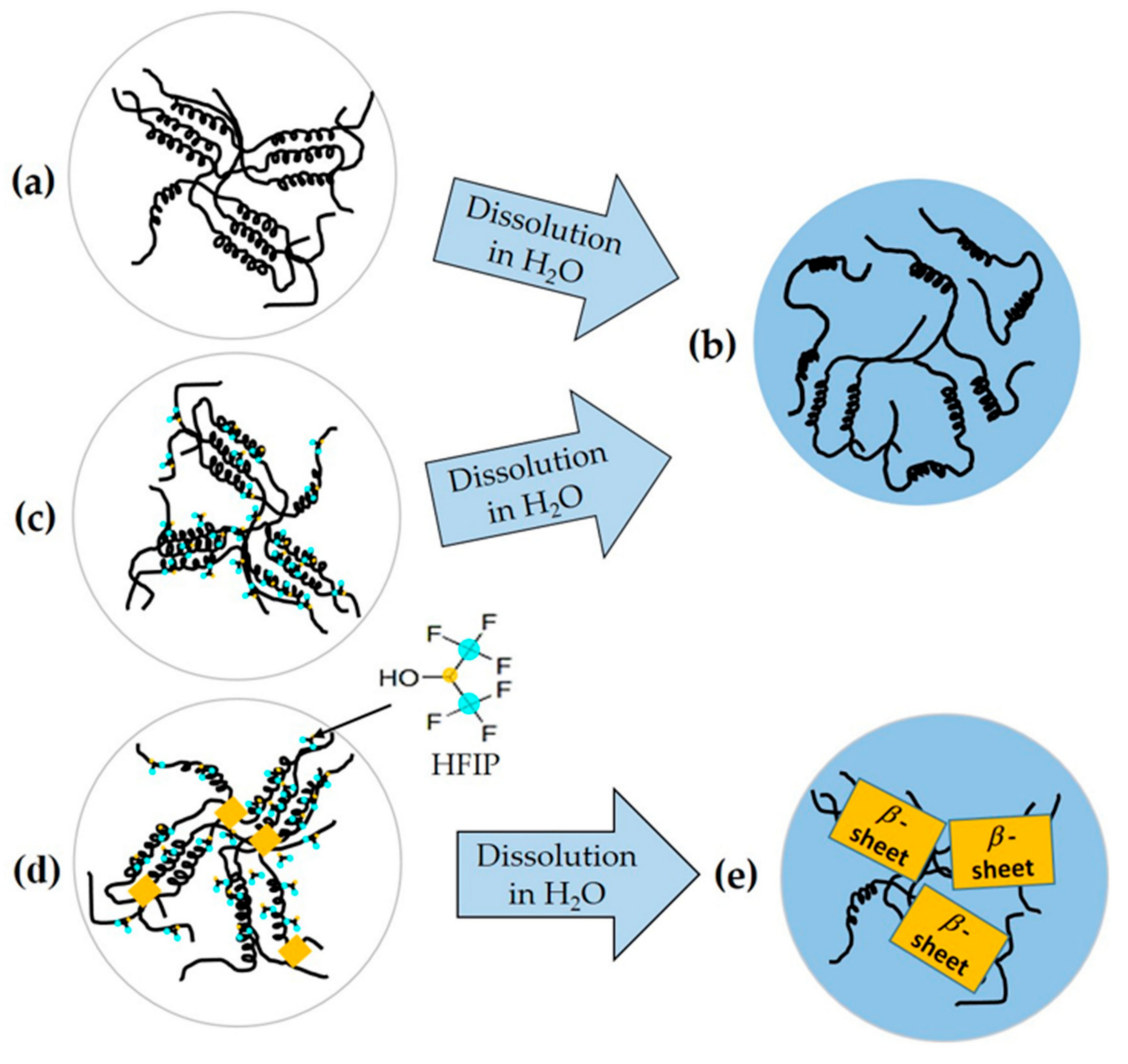
The LF_aq_ as-cast film has an ordered aggregated *α*-helix structure (**a**) that easily dissolves in water (**b**). When treated with HFIP gas, the aggregated *α*-helices in the LF_aq_ as-cast film and HFIP molecules bind. However, due to low molecular movement in the solid state, the overall aggregation state of the *α*-helices is retained (**c**). The bound HFIP is easily removed by water, resulting in dissolution of the film. On the other hand, the 40%-EtOH-treatment induces imperfect *β*-sheet formation in the SGF-gel. As a result, the SGF-gel PSG is soluble in HFIP. However, some *β*-sheet structural memory remains, and the SGF_HFIP_ as-cast film cast from a solution of the SGF-gel in HFIP has some *β*-sheet content which renders it water-resistant (**d**). Further, removal of residual HFIP in the SGF_HFIP_ as-cast film by water renders it *β*-sheet-rich (**e**), indicating an occurrence of water induced *β*-sheet formation.

The current results reveal a structure water-solubility relationship in the cast films whose mechanism is summarized in Figure 8. In the LF_aq_ as-cast film, the interactions between the ordered aggregated *α*-helices (Figure 8a) are weak, and easily dissolve in water (Figure 8b). When this film is treated with HFIP gas, the HFIP molecules and the *α*-helices bind, but the molecular movements in the solid-state are relatively low and the “overall” aggregation state of the *α*-helical domains is retained (Figure 8c). On being placed on a water surface, HFIP molecules easily dissolve due to their high affinity for water. As a result, the film treated with HFIP, now similar to the LF_aq_ as-cast film, dissolves in water. On the other hand, the SGF_HFIP_ as-cast film has some *β*-sheet content (indicated by yellow boxes in Figure 8d) that results from some *β*-sheet structural memory induced by treatment of SGF with 40%-EtOH. This renders the SGF_HFIP_ as-cast film water-stable.

**Figure 9:**
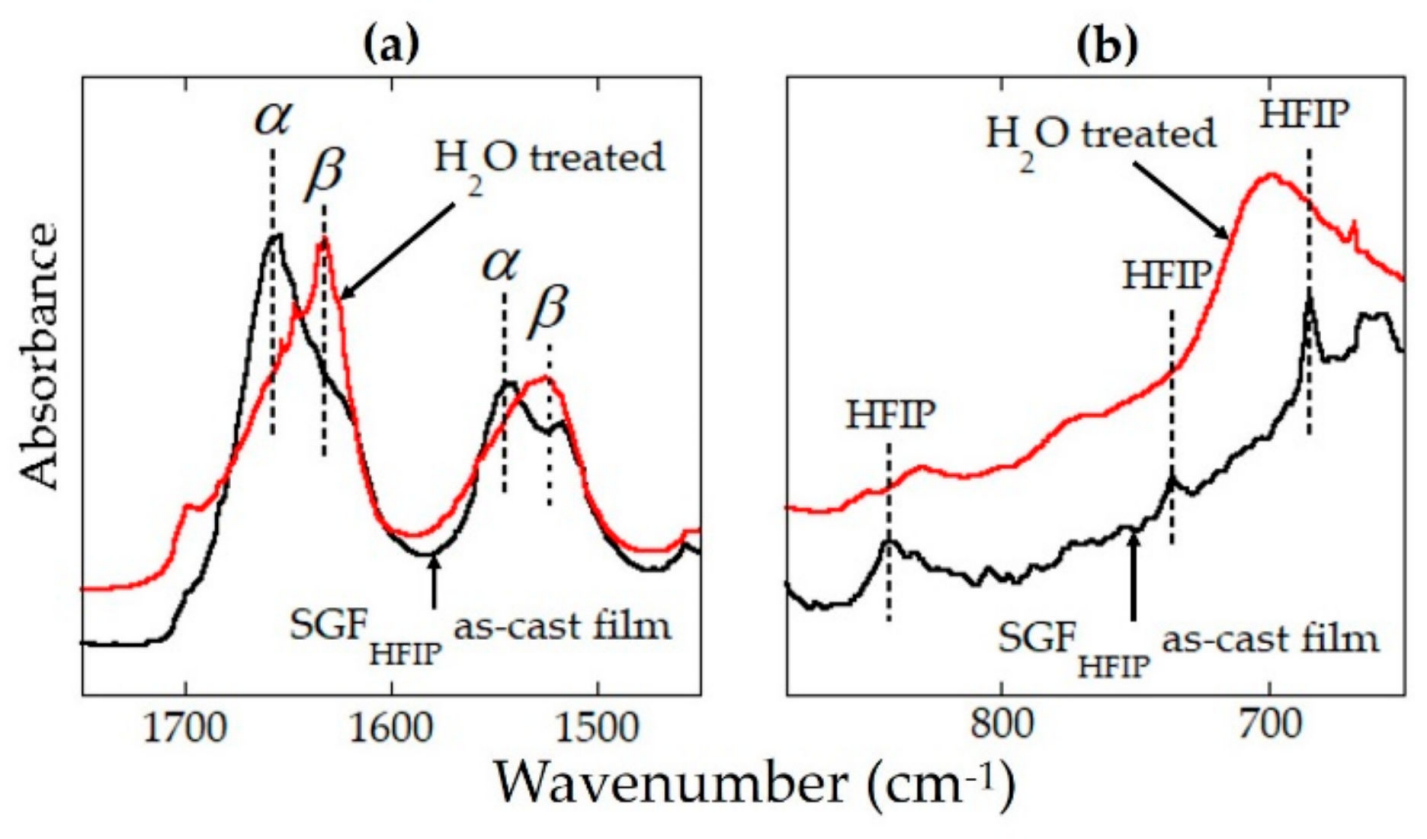
FTIR spectra of *S. c. ricini* as-cast (spectrum in black) and water-treated (spectrum in red) SGF_HFIP_ films. The vibrational bands in the amide I and II regions are shown in panel (**a**), whereas those assigned to HFIP are shown in panel (**b**).

For use as a biomaterial, residual HFIP in the SGF_HFIP_ as-cast film must be removed. Kameda et al. reported successful removal of residual HFIP from a film cast from hornet silk by rinsing in water [24]. To evaluate if this strategy could be applicable in the current study, the SGF_HFIP_ as-cast film was immersed in water for 12 h and its structural properties in the dry state were characterized by FTIR. FTIR spectra of the as-cast and water-treated SGF_HFIP_ films were as shown in Figure 9. Owing to its high affinity for water, residual HFIP dissolved when the SGF_HFIP_ as-cast film was immersed in water, as confirmed by the disappearance of the peaks assigned to HFIP (Figure 9b). The now-HFIP-free SGF_HFIP_ film, thought to be similar to the LF_aq_ as-cast film, did not dissolve in water, instead, a shift to higher wavenumbers of the amide I and II vibrational bands was detected in its FTIR spectrum in the dry state. This indicates an occurrence of water induced *β*-sheet formation from the aggregated *α*-helix structure (Figure 9a). *β*-sheet formation is well known to induce water-resistance in SF-based materials [25], hence the difference in water resistance between the LF_aq_ and SGF_HFIP_ as-cast films. Moreover, structural change, back to the *α*-helical conformation, attributed to preferences of the polyalanine domains in hydrophobic and hydrophilic environments, respectively, upon drying of water-treated artificial spidroins has been reported [26,27]. However, the dominant structure of the water-treated SGF_HFIP_ film in the wet state remains unclarified and will be of interest in our future investigations.

### 2.4. Superior Wet-Drawability of the SGF_HFIP_ As-Cast Film

Solvents have been reported to influence some properties, especially the mechanical properties, of SF films [28]. It is also well documented that drawing results in *α*-helix to *β*-sheet conformation transition in various types of silks [6,29,30,31,32]. With this understanding, and the *β*-sheet formation that occurs when the SGF_HFIP_ as-cast film is immersed in water (discussed in the preceding section), possible slipping of the *α*-helical chains during drawing is expected to be suppressed. This implies that the applied tension would be transmitted uniformly throughout the film. Therefore, we hypothesized that the SGF_HFIP_ as-cast film could be wet-drawn (while immersed in water). Moreover, since drawing is accompanied by a transition from the compact helical conformation to the elongated chain (*β*-sheet) structure, the films’ maximum draw-ratio (DR) could be higher. Theoretically, the distance separating each complete turn along the helix axis is 5.4 Å (1.5 Å per residue), whereas a fully extended *β*-sheet structure corresponds to 3.4 Å per residue. Thus, a theoretical DR in changing from *α*-helix to *β*-sheet would be ~2.27. To confirm these hypotheses, SGF_HFIP_ and LF_aq_ as-cast films treated with 80%-EtOH, to induce *β*-sheet formation, were drawn alongside the as-cast SGF_HFIP_ film. Drawability, expressed as strain at break (%), attained for the films was as presented in Table 2.

**Figure 10:**
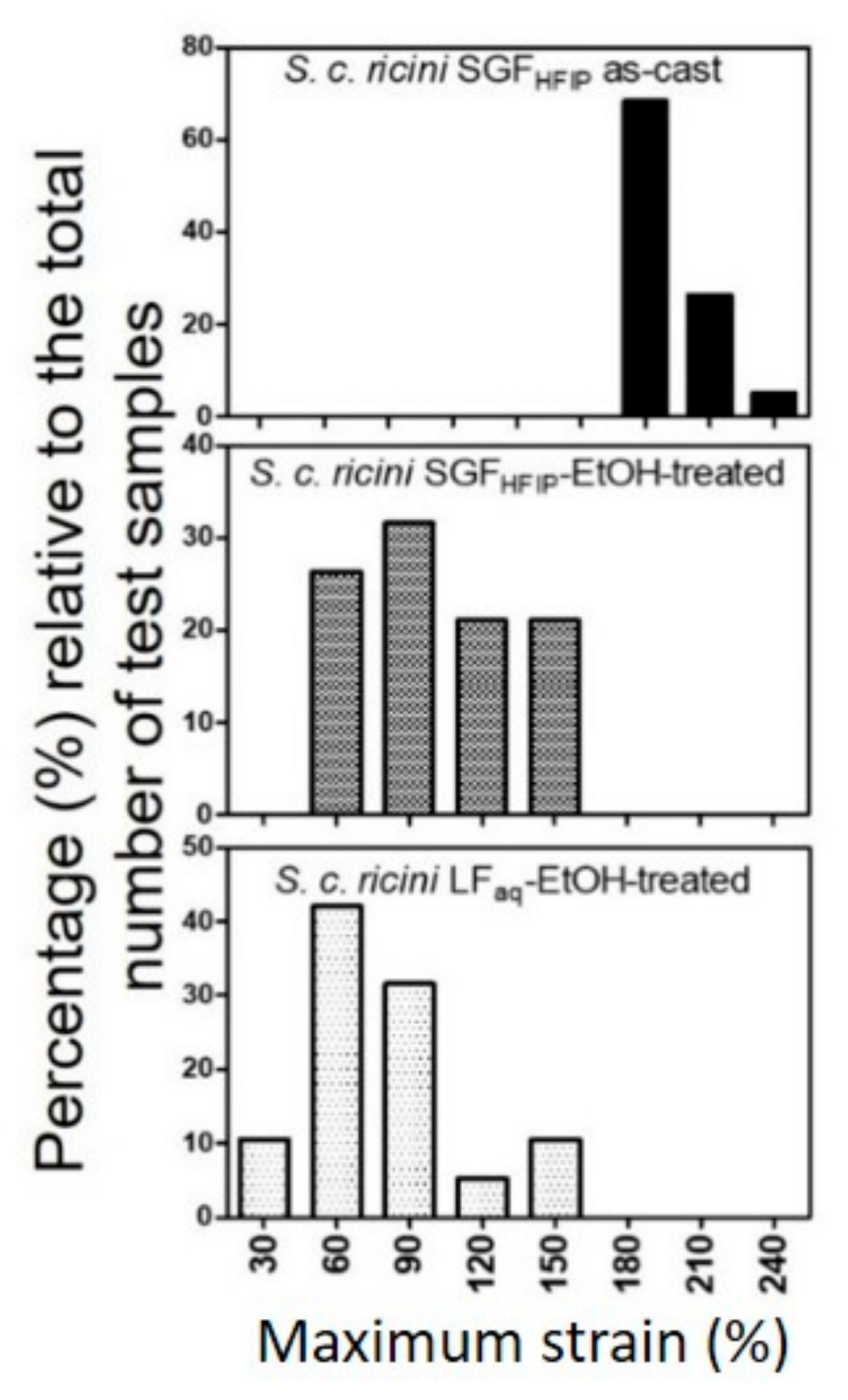
Relative frequency distribution of the maximum strain (%) of the as-cast SGF_HFIP_, and 80%-EtOH SGF_HFIP_ and LF_aq_ films.

The relative frequency distribution (%) of the maximum strain (strain at break) of the three kinds of SF films were as shown in Figure 10. Approximately 90% of the time, the SGF_HFIP_ as-cast film could be successfully drawn to a DR >3 (corresponding to a maximum stain of 200%), whereas the 80%-EtOH-treated LF_aq_ and SGF_HFIP_ films could be drawn to maximum DR less than 2 (corresponding to a maximum strain of 100%). This was because the SGF_HFIP_ as-cast film was initially *α*-helix-rich, whereas the EtOH-treated LF_aq_ and SGF_HFIP_ films were *β*-sheet-rich, further supporting our interpretation. We interpret the *α*-helix to *β*-sheet conformation transition during wet-drawing of the SGF_HFIP_ cast film to be due to (1) stretching and (2) α-helix to *β*-sheet conformation transition. *β*-sheet crystallization decreases drawability of the SGF_HFIP_ as-cast film, and the maximum DR achieved includes such a suppression effect, although other factors that may influence wet-drawability of the film such as film thickness, temperature, and drawing rate, are yet to be clarified. In earlier studies, drawing of semi-dried *S. c. ricini* silk dope gave a considerably high DR (~10) [6,29]. This is because the drawing force may break the interchain bonds resulting in extension and even slipping of the helical chains. Taking our results and the theoretical interpretation into account, slipping of molecular chains during drawing may largely account for the considerably high DR in earlier studies.

### 2.5. Industrial Prospects of the Current Fabrication Strategy

*S. c. ricini* silk glands are thin and flexuous, and peeling of the gland epithelium was time consuming. Due to these, further assessment was done on the possibility of obtaining a HFIP solution of SGF from the 40%-EtOH-treated PSG without peeling (see Table 1, SGF_HFIP_, b). Portions of the 40%-EtOH-treated PSG were directly immersed in HFIP and the dissolved SGF was separated from the undissolved gland epithelium by filtration. As shown in Table 1, Ser residues in a film cast from the SGF-HFIP solution obtained (SGF_HFIP_, b) were comparable, or even less than those in the conventional LF_aq_ as-cast film, indicating better separation of the SGF-gel and gland epithelial layer. We expect that the application of this strategy will be useful in obtaining HFIP solutions of *S. c. ricini* SGF in an industrial scale.

## 3. Experimental Section

### 3.1. Fabrication of Cast Films

*S. c. ricini* silkworm larvae were reared in the laboratory on an all-instar artificial diet, SilkMate L4M (Nosan Corp., Yokohama, Japan) at 25 ± 3 °C and a relative humidity of 65 ± 3%, as earlier described [12]. Late fifth instar larvae were dissected to obtain intact silk glands. The glands were carefully rinsed in Milli-Q water, immersed in 40% v/v aqueous ethanol (40%-EtOH) (Wako Pure Chemical Ltd, Osaka, Japan) to induce gelation of fibroin [13], and kept at 4 °C. Gelation of the silk dope was visually observed to occur after about 3 h under these conditions. The silk gland is longitudinally differentiated into functionally distinct posterior (PSG), middle (MSG), and anterior (ASG) sections [33]. The PSG, where fibroin is synthesized and stored as a weak gel before spinning, was separated from the middle and anterior sections, and its epithelium was carefully peeled to obtain the silk gland fibroin gel (SGF-gel). The SGF-gel obtained was dissolved in neat HFIP (99.0%; Tokyo Chemical Industry Co., Ltd, Tokyo, Japan) in sealed screw cap tubes under mild agitation at 23 ± 2 °C. However, the PSG of *S. c. ricini* are thin and flexuous and long durations would be required to obtain workable amounts of SGF-gel. Here, we hypothesize that if only the SGF-gel would dissolve in HFIP when whole (unpeeled) 40%-EtOH-treated PSGs are dissolved, then by physical separation of the dissolved fraction, it could be possible to obtain a solution of SGF in HFIP. To test this hypothesis, about 2.0 cm portions of the unpeeled, 40%-EtOH-treated PSG were dissolved in HFIP under mild agitation at room temperature. The SGF-gel in the gland lumen dissolved within several hours (~2 h) and the undissolved epithelium was separated from the fibroin solution by filtration.

For comparison, fibroin from the PSG of *S. c. ricini* was gently drained into cold Milli-Q water and rinsed by exchanging the water at least thrice to remove any contaminants as previously described [12], and diluted with Milli-Q water to give a transparent solution of aqueous LF (LF_aq_). Films were prepared by separately casting the SF solutions onto polystyrene Petri dishes. After drying at 23 ± 2 °C and a relative humidity of 30 ± 2%, transparent and flexible films that could be peeled off the substrate were obtained. The thickness of the cast films was controlled by adjusting the concentration or volume of the fibroin solutions cast. Moreover, from each batch of *S. c. ricini* larvae reared, some were let to spin cocoons. These were cut open by a pair of scissors to remove the pupae and, for all analyses, portions of the native fiber were sampled from the middle section of the middle layer of the fresh cocoons.

The as-cast films and native fiber were heat-, HFIP-gas-, water-, and EtOH-treated and used for the respective analyses as described in Section 3.4 through Section 3.8 For heat treatment, rectangular portions of the LF_aq_ and SGF_HFIP_ as-cast films and native fiber were were sandwiched (separately) between thin glass slides (~1.0 mm thick) alongside a sensitive thermocouple to monitor the sample temperature. These were then heated on a digital hotplate (Corning PC-420D) to 220 °C and kept at this temperature (annealed) for 1 min. For HFIP-gas-treatment, LF_aq_ as-cast films were placed in a container saturated with HFIP gas at 23, 30, 40, and 50 °C for 0.3, 3/2, 1, 2, 3, 4, 5, and 6 h to obtain various HFIP-gas-treated films. For water treatment, portions of the SGF_HFIP_ as-cast films were immersed in water for 12 h and dried at room temperature. Lastly, for EtOH treatment, the LF_aq_ and SGF_HFIP_ as-cast films were immersed in aqueous 80%-EtOH for 12 h and dried at room temperature.

### 3.2. Determination of Molecular Weight (Mw) 

Mw distribution of the SGF-gel and LF_aq_ as-cast film were determined by gel permeation chromatography (GPC). For each measurement, about 20 mg of the fibroin protein sample was dissolved in hexafluoroacetone trihydrate at 23 ± 2 °C for 24 h. The concentration of the solution was adjusted to about 0.02 wt.% with the eluent (HFIP) containing 1.0 mL of 5.0 mM sodium trifluoroacetate and filtered using a 5.0 μm polytetrafluoroethylene (PTFE) filter syringe. The chromatographic set-up comprised of a HLC-8320GPC system (Tosoh Corp., Tokyo, Japan) fitted with a 4.6 mm i.d. × 3.5 cm, 4 μm TSKgel SuperH-H guard column (Tosoh Corp., Tokyo, Japan), two (6.0 mm i.d. × 15 cm, 3.0 and 5.0 μm, TSKgel SuperHM-H separation columns (Tosoh Corp., Tokyo, Japan), and a dual flow refractive index (RI) detector (Tosoh Corp., Tokyo, Japan). The standard M_W_ markers were polymethyl methacrylate (PMMA) in the range 0.55 to 2,100 kDa.

### 3.3. Amino Acid Composition Analysis

The amino acid compositions of the LF_aq_ and SGF_HFIP_ as-cast films were evaluated by high performance liquid chromatography (HPLC). For each measurement, about 5.0 mg of the fibroin protein was hydrolysed in 6 M HCl at 110 ℃ for 22 h and dried with a N_2_ purge. The hydrolysates were dissolved in 500 μL of citric acid buffer (pH 2.2), and de-proteinized by centrifugal filtration (Ultrafree-MC 0.45 μm, 13,400 rpm, 2 min). An analytical blank was similarly prepared. Each filtrate was appropriately diluted and analysed by a post-column derivatization with *o*-phthalaldehyde (OPA) and N-acetyl-L-cysteine using a HPLC system (Shimadzu, LC-VP) equipped with a Shim-pack Amino-Na (100 mm L × 6.0 mm i.d.) cation exchange column (Shimadzu, Japan) and an ISC-30/S0504 Na (50 mm L × 4.0 mm i.d.) ammonia trap column. A gradient elution was performed in the high-pressure mode using sodium citrate buffers (A) and (B) and sodium hydroxide (C) as mobile phases at a flow rate of 0.4 mL min^−1^. The column temperature and injection volume were 60 °C and 10 µL, respectively. A Shimadzu RF-20A*_XS_* fluorescence detector was used to detect the fluorescence products at excitation and emission wavelengths of 350 and 435 nm, respectively. Tenfold dilutions of the amino acid mixture standard solution type H (Wako Pure Chemical Industries Ltd., Osaka, Japan) were analysed to obtain a calibration curve used for quantitation of each component. 

### 3.4. Fourier Transform Infrared (FTIR) Spectroscopy

FTIR measurements of the as-cast, and heat-, HFIP-gas-, water-, and EtOH-treated LF_aq_ and SGF_HFIP_ films were recorded at room temperature with an FTIR-620 (JASCO International Co. Ltd., Japan) spectrometer in the transmission mode. For each measurement, 32 scans were co-added in the spectral range 4000 to 400 cm^−1^ with a 2 cm^−1^ resolution. The resultant spectrum was corrected by subtracting a background spectrum recorded under similar scan conditions. The assignments of conformation sensitive bands for *S. c. ricini* SF and HFIP used in this report were similar to those in our earlier study [12]: ~1625 and ~1519 cm^−1^ as *β*-sheet structure [34,35], ~1658 and 1545–1548 cm^−1^ as *α*-helix [10,34], and ~894, ~736, and ~686 cm^−1^ as characteristic peaks assigned to HFIP [16].

### 3.5. ^13^C Cross-Polarization Magic-Angle Spinning Solid-State NMR Spectroscopy

The ^13^C cross-polarization magic-angle spinning solid-state NMR measurements of native fiber, and the LF_aq_ and SGF_HFIP_ as-cast films were carried out with a Bruker Avance 600 WB (Karlsruhe, Germany) spectrometer with a magnetic field of 14.1 T at room temperature. The spectrometers were operated at a ^13^C NMR frequency of 150.94 MHz. For each measurement, the fibroin protein sample was cut into small pieces, packed into a solid-state probe, and spun at a MAS frequency of 10.0 kHz in a 4.0 mm Ø zirconia rotor sample tube. A ^1^H 90° pulse length of 3.5 μs and ^1^H-^13^C cross-polarization (CP) contact of 70 kHz were employed for the CP experiments. High-power ^1^H decoupling using the SPINAL-64 method was employed. The CP experiments were repeated every 3.0 s and the ^13^C chemical shifts were calibrated externally through the adamantane methylene peak at 29.5 ppm relative to TMS at 0 ppm.

### 3.6. Thermal Analyses

Differential scanning calorimetry (DSC) measurements of native fiber and heat-, HFIP-gas-, water-, and EtOH-treated LF_aq_ and SGF_HFIP_ films were performed with a DSC Q200 analysis system (TA Instruments, USA). Instrument calibration for heat flow and temperature were done using indium, whereas for heat capacity, aluminum and sapphire were used. For each measurement, about 5.0 mg of the fibroin protein sample was encapsulated in an aluminum pan and heated between −30 and 430 °C at 2 °C min^−1^ under a dry nitrogen nitrogen gas flow of 50 mL min^−1^.

The weight loss profiles of the the SF solutions, native fiber and heat-, HFIP-gas-, water-, and EtOH-treated LF_aq_ and SGF_HFIP_ films were determined by thermal gravimetry (TG) with a Thermoplus TG 8120 system (Rigaku Corp., Tokyo, Japan). For each measurement, about 1.0 mg of the fibroin protein sample was heated in an aluminium pan from room temperature to 430 °C at a heating rate of 2 °C min^−1^ under a dry nitrogen gas flow of 200 mL min^−1^.

### 3.7. Wide-Angle X-Ray Diffraction Analyses

WAXD measurements of native fiber and heat-, HFIP-gas-, water-, and EtOH-treated LF_aq_ and SGF_HFIP_ films were carried out with a 3.5 m NANOPIX X-ray diffractometer (Rigaku Corp., Tokyo, Japan) (40 kV, 30 mA, Cu−K_α_ radiation, λ = 1.5418 Å) equipped with a highly sensitive single-photon counting pixel HyPix-6000 2D detector with a pixel size of 100 × 100 µm^2^ (Rigaku Corp., Tokyo, Japan), as previously described [36]. Calibration of the camera distance was carried out using cerium oxide powder and a background diffraction pattern collected under conditions similar to those used for sample analysis was subtracted from each measurement. The crystallite sizes were evaluated on the basis of the Scherrer equation.

### 3.8. Wet-Drawing of the Cast films

Wet-drawability of as-cast SGF_HFIP_, and 80%-EtOH-treated SGF_HFIP_ and LF_aq_ films was evaluated by manually drawing rectangular strips (2 × 10 mm) of the films using a hand-held stretching device while immersed in water (wet-drawing) at room temperature until break (maximum DR; ratio of the length of the film at break and before drawing). The diameter of the strips was ~20 µm and 19 replicate samples for each of the three kinds of test films were drawn.

## 4. Conclusions

We successfully prepared a transparent, flexible, *α*-helix-rich, and water-resistant as-cast film without post-treatment. By use of aqueous 40%-EtOH, *S. c. ricini* silk gland fibroin (SGF) was solidified and separated from the rest of the silk gland. The SGF-gel dissolved in HFIP at room temperature within several hours, implying that it has an imperfect *β*-sheet structure. However, some *β*-sheet memory remained and a film cast from this SGF-HFIP solution (SGF_HFIP_ as-cast film) had some *β*-sheet structure, which rendered it water resistant. In water, residual HFIP dissolves and the film becomes *β*-sheet-rich, indicating an occurrence of water-induced *β*-sheet formation. These results reveal an important structure water-solubility relationship in the *α*-helix-rich films cast from *S. c. ricini* SF. Based on these, a plausible model of the mechanism leading to the difference in water resistance of the LF_aq_ and SGF_HFIP_ as-cast films is proposed. This water-resistant film is relatively easy to fabricate and may be attractive as a biomaterial. Further, we expect this fabrication strategy to be applicable to the other kinds of wild silkworm silk.

## Figures and Tables

**Table 1:** Amino acid compositions (mol %) of films cast from aqueous liquid fibroin (LF_aq_) and 1,1,1,3,3,3-hexafluoro-2-propanol (HFIP) solutions of the 40%-EtOH-treated posterior silk gland (PSG) SGF_HFIP_ with (**a**) and without (**b**) peeling the gland epithelium.

Amino Acid	LF_aq_	SGF_HFIP_ (a)	SGF_HFIP_ (b)
Gly	33.8	34.5	34.9
Ala	44.2	40.0	41.3
Pro	0.4	0.5	0.5
Val	0.5	0.5	0.6
Leu	0.4	0.5	0.5
Ile	0.4	0.4	0.4
Met *	*	*	*
Ser	6.1	7.2	5.6
Thr	0.5	0.8	0.7
Cys *	*	*	*
Asx **	3.7	4.0	4.0
Glx **	0.9	1.0	1.1
Arg	1.8	2.1	2.1
Lys	0.4	0.5	0.5
His	1.6	1.8	1.9
Tyr	5.3	6.3	5.9
Phe	0.3	0.2	0.2
Trp #	-	-	-
**Gly/Ala**	**0.8**	**0.9**	**0.9**
**Acidic**	**4.6**	**5.0**	**5.1**
**Basic**	**3.8**	**4.3**	**4.5**

* Met and Cys were below the quantitation limit of the current analytical method. Further, under conventional acid hydrolysis, # Trp is generally not recovered and ** Asn and ** Gln are de-aminated to Asp and Glu, respectively.

**Table 2:** Strain at break (mean ± SD; %) of the as-cast SGF_HFIP_ and 80%-EtOH-treated SGF_HFIP_ and LF_aq_ films (n = 19).

Sample	Strain at Break (%)		
	Minimum	Maximum	Average
SGF_HFIP_ as-cast film	180	230	202 ± 14
80%-EtOH-treated SGF_HFIP_ cast film	40	160	100 ± 33
80%-EtOH-treated LF_aq_ cast film	60	150	79 ± 29

## Supplementary Materials

The following are available online; Figure S1, differential Mw distributions of the SGF-gel (curve in red) and LF_aq_ as-cast film (curve in black); Figure S2, FTIR spectra of SGF_HFIP_ films cast from solutions prepared by dissolution of *S. c. ricini* SGF-gel in HFIP at 23 ± 2 °C for 1.25, 2, 8, and 24 h; Figure S3, a transparent and flexible SGF_HFIP_ film cast from a solution obtained by dissolution of the SGF-gel in HFIP at 23 ± 2 °C for 12 h; Figure S4, water solubility of *S. c. ricini* LF_aq_ (a) and SGF_HFIP_ (b) as-cast films; Figure S5, FTIR spectra of *S. c. ricini* LF_aq_ and SGF_HFIP_ as-cast films showing the peaks assigned to HFIP; Figure S6, DSC *β*-sheet crystallization profiles of the HFIP-gas-treated LF_aq_ film at 30 (a) and 40 (b) °C for 1, 2, 3, 4, 5, and 6 h. The panels on the right are plots of the *β*-sheet crystallization peak temperatures against the duration of exposure to HFIP gas.
