# Supplementary material for: Structure Water-Solubility Relationship in α-Helix-Rich Films Cast From Aqueous and 1,1,1,3,3,3-Hexafluoro-2-Propanol Solutions of S. c. ricini Silk Fibroin
Source: Molecules. 2019 Oct 31;24(21):3945. doi: 10.3390/molecules24213945 (PMC6864477; doi:10.3390/molecules24213945)
Supplement: Supplementary file 1 [file molecules-24-03945-s001.pdf]

Supplementary Information for:

Structure Water-Solubility Relationship in *α*-Helix-Rich Films Cast From Aqueous and 1,1,1,3,3,3-Hexafluoro-2-Propanol Solutions of *S. c. ricini* Silk Fibroin

Kelvin O. Moseti [1,2,3], Taiyo Yoshioka [2], Tsunenori Kameda [2,*] and Yasumoto Nakazawa [1,*]

1 Department of Biotechnology and Life Science, Graduate School of Engineering, Tokyo University of Agriculture and Technology, 2-24-16 Naka-cho, Koganei, Tokyo 184-8588, Japan; kelvinmoseti@gmail.com
2 Silk Materials Research Unit, Institute of Agrobiological Sciences, National Agriculture and Food Research Organization, 1-2 Owashi, Tsukuba, Ibaraki 305-8634, Japan; yoshiokat@affrc.go.jp
3 National Sericulture Research Centre, Industrial Crops Research Institute, Kenya Agricultural and Livestock Research Organization, P.O. Box 7816-01000, Thika, Kenya
* Correspondence: kamedat@affrc.go.jp (T.K.); yasumoto@cc.tuat.ac.jp (Y.N.); Tel.: +81-29-838-6213 (T.K.); +81-42-388-7612 (Y.N.)

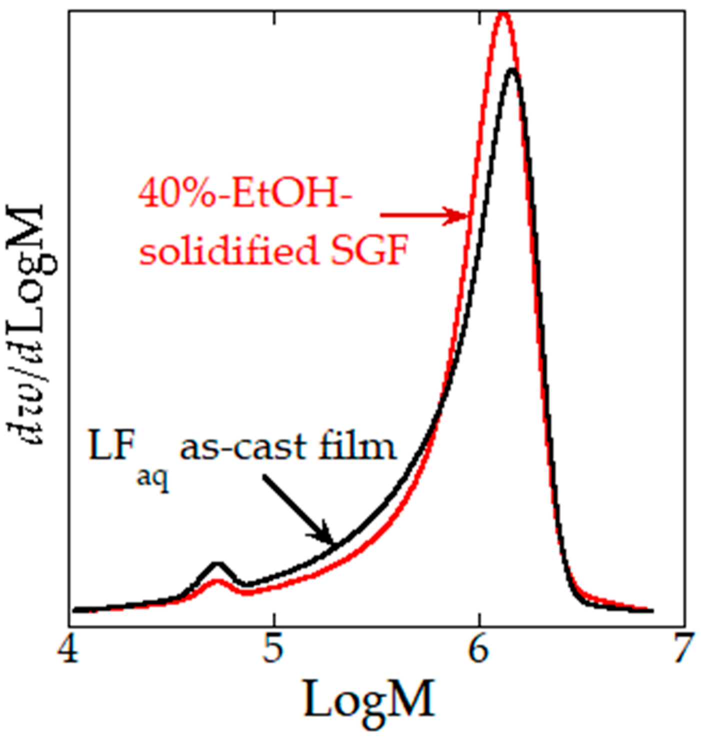

Figure S1. Differential Mw distributions of *S. c. ricini* SGF-gel (curve in red) and LF_{aq} as-cast film (curve in black).

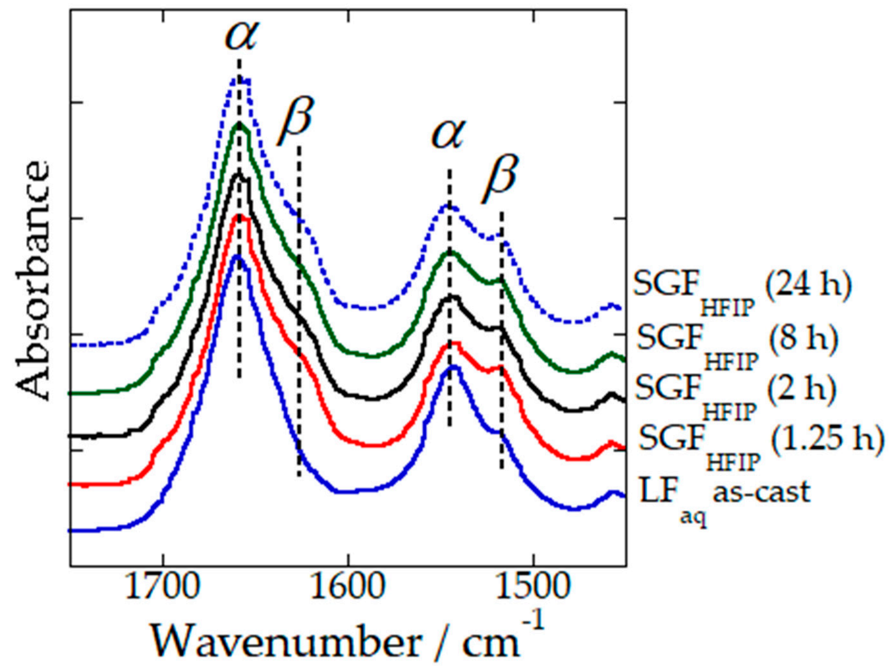

Figure S2. FTIR spectra of SGF_{HFIP} films cast from solutions prepared by dissolution of *S. c. ricini* SGF-gel in HFIP at 23 ± 2 °C for 1.25, 2, 8, and 24 h.

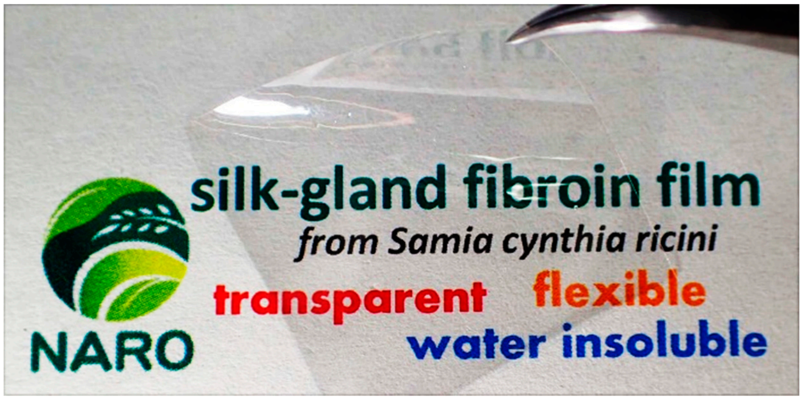

Figure S3. A transparent and flexible film (SGF_{HFIP}) cast from a solution obtained by dissolution of *S. c. ricini* SGF-gel in HFIP at 23 ± 2 °C for 12 h.

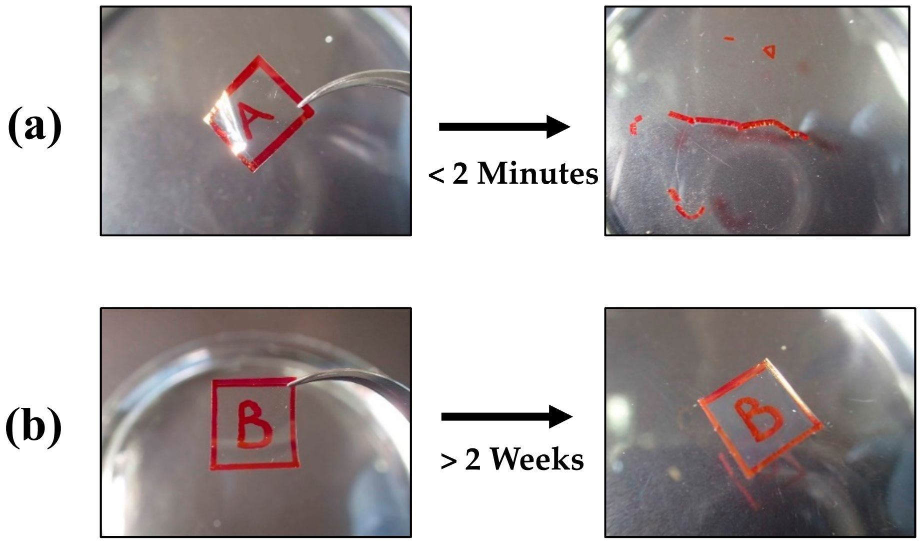

Figure S4. Water solubility of *S. c. ricini* LF_{aq} (a) and SGF_{HFIP} (b) as-cast films.

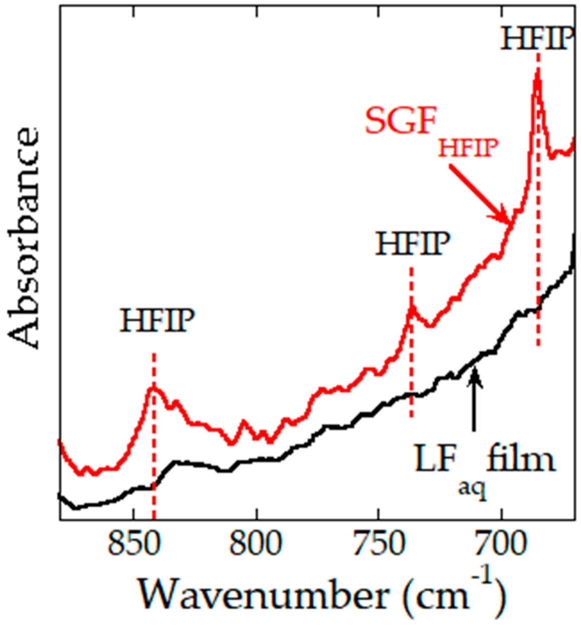

Figure S5. FTIR spectra of *S. c. ricini* LF_{aq} and SGF_{HFIP} as-cast films showing the peaks assigned to HFIP.

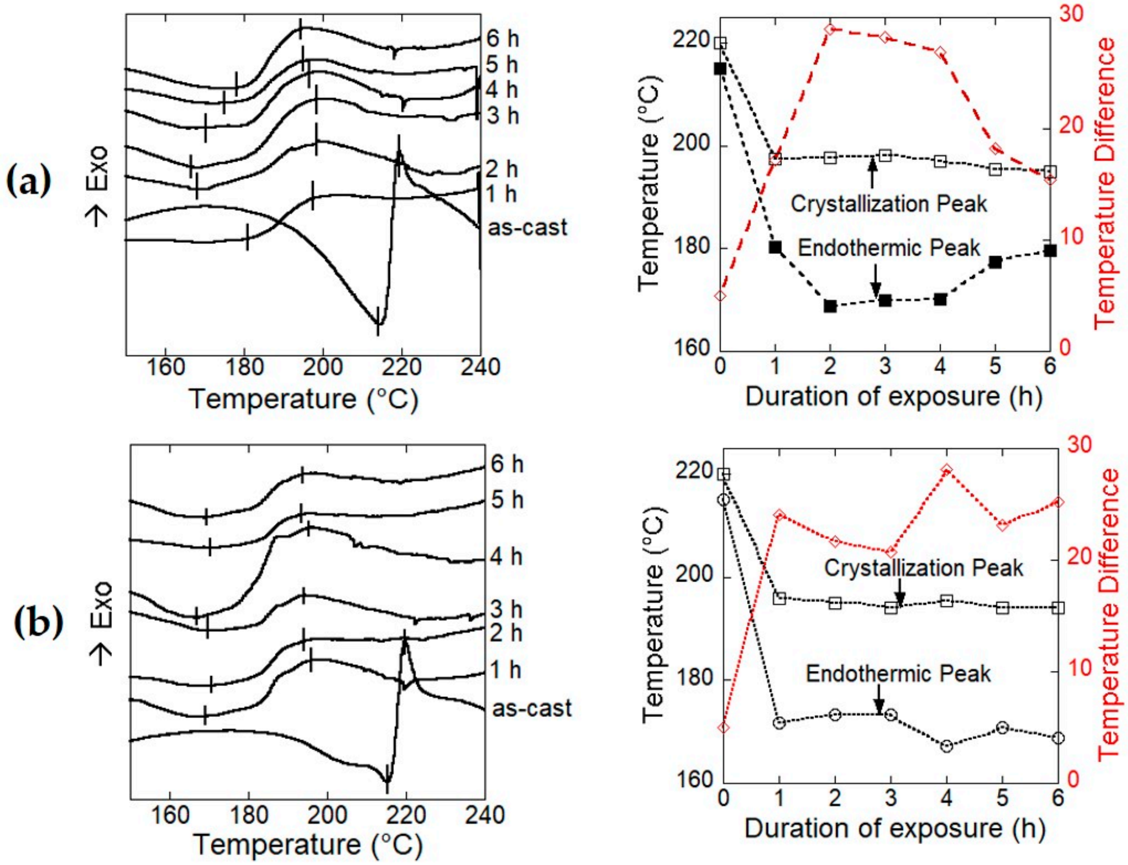

Figure S6. DSC *β*-sheet crystallization profiles of *S. c. ricini* LF_{aq} film treated with HFIP gas at (**a**) 30 and (**b**) 40 °C for 1, 2, 3, 4, 5 and 6 h. The panels on the right are plots of the *β*-sheet crystallization peak temperatures against the duration of exposure to HFIP gas.
